# Prognostic Value of Conventional and Modified Naples Scores for In-Hospital Mortality in Patients with Non-ST-Elevation Acute Coronary Syndrome

**DOI:** 10.3390/medicina62081573

**Published:** 2026-08-17

**Authors:** Yusuf Cekici, Abdullah Yildirim, Emre Pacaci, Mükremin Coskun, Emre Sezici, Hasan Burak Ozdemir, Sefa Sural, Ozgun Gulaydin, Fadime Koca, Omer Bedir, Murat Gencaslan, Ceyhun Yucel, Mehmet Kucukosmanoglu

**Affiliations:** 1 Department of Cardiology, Adana City Training and Research Hospital, University of Health Sciences, 01230 Adana, Turkey; mukremincoskun@gmail.com (M.C.); emresezici@gmail.com (E.S.); hsn.burakozdemir@gmail.com (H.B.O.); ozgungulaydin2121@hotmail.com (O.G.); dromerbedir@gmail.com (O.B.); dr.muratgencaslan@gmail.com (M.G.); dr.ceyhunyucel01@gmail.com (C.Y.); mehmetkoo@yahoo.com (M.K.); 2 Department of Cardiology, Cukurova State Hospital, 01170 Adana, Turkey; dr.yildirimabdullah@gmail.com (A.Y.); drfadimekoca@gmail.com (F.K.); 3 Department of Cardiology, Osmaniye Training and Research Hospital, University of Health Sciences, 80010 Osmaniye, Turkey; emre.pcci@hotmail.com; 4 Vocational School of Health Service, Toros University, 33140 Mersin, Turkey; drsefasural@yahoo.com

**Keywords:** Naples score, C-reactive protein, modified Naples prognostic score, Naples prognostic score, non-ST-elevation acute coronary syndrome

## Abstract

*Background and Objectives*: Inflammation and nutritional status are closely associated with prognosis in patients with non-ST-elevation acute coronary syndrome (NSTE-ACS). This study compared the prognostic performance of the conventional Naples score and CRP-modified Naples score for predicting in-hospital mortality. *Materials and Methods*: This retrospective, single-center study included 921 patients with NSTE-ACS undergoing percutaneous coronary intervention. Multivariable logistic regression, ROC analysis, reclassification indices, restricted cubic spline analysis, and decision curve analysis were performed. *Results*: In-hospital mortality occurred in 79 patients (8.6%). Patients who died had higher Naples score and modified Naples score values than survivors. In multivariable models adjusted for baseline clinical variables and GRACE score, both the modified Naples score and conventional Naples score remained independently associated with in-hospital mortality. The modified Naples score showed an adjusted-OR of 3.85 (95% CI: 2.71–5.48; *p* < 0.001), whereas the conventional Naples score showed an adjusted-OR of 4.52 (95% CI: 3.05–6.70; *p* < 0.001). The addition of the modified Naples score improved model performance, with a C-index of 0.819, NRI of 0.741, and IDI of 0.127. Similarly, the conventional Naples score model showed a C-index of 0.827, NRI of 0.843, and IDI of 0.125. In ROC analysis, the AUC was 0.701 for the modified Naples score and 0.683 for the conventional Naples score, without a statistically significant difference between them (ΔAUC = 0.017; *p* = 0.300). Decision curve analysis showed positive net clinical benefit for both scores, with no consistent superiority of either score. *Conclusions*: Both Naples-based scores were independently associated with in-hospital mortality and provided comparable prognostic performance in patients with NSTE-ACS undergoing PCI.

## 1. Introduction

Atherosclerosis is a complex process driven by the activation of multiple cascades, among which chronic, systemic, low-grade inflammation is considered one of the key underlying mechanisms [1,2]. An imbalance between pro- and anti-atherogenic pathways can consequently give rise to the clinical manifestations of atherosclerosis, including coronary artery disease (CAD), cerebrovascular disease, and acute myocardial infarction (AMI) [3,4,5].

Although inflammatory status can be assessed through various methods, serum-derived parameters remain among the most accessible and practical. Prior studies have shown that serum markers such as neutrophils, lymphocytes, monocytes, C-reactive protein (CRP), albumin, and uric acid are linked to adverse outcomes in patients with chronic or acute coronary syndrome (ACS) [6,7,8]. The Naples Score, known as the Naples Prognostic score—comprising serum albumin, total cholesterol, neutrophil/lymphocyte ratio (NLR), and lymphocyte/monocyte ratio (LMR)—has shown prognostic value across a range of cardiovascular, cerebrovascular, renal, oncologic, and endocrinologic conditions [9,10,11,12,13,14,14]. However, due to certain limitations, several modified and validated versions of the score have since been proposed. While some of these modifications focused on revalidating cutoff values, more recent studies have reported that a CRP-modified version offers greater clinical utility specifically in cardiovascular disease [13,15].

This study aims to evaluate these relationships in patients with non-ST-elevation ACS (NSTE-ACS) undergoing percutaneous coronary intervention (PCI), in order to determine the risk of in-hospital all-cause mortality using a modified Naples score incorporating CRP, and to compare its performance with other inflammatory indices, particularly the conventional Naples score.

## 2. Materials and Methods

### 2.1. Study Design and Setting

This retrospective, single-center observational study was conducted between January 2023 and February 2024. A total of 1650 consecutive patients admitted to the coronary intensive care unit with a diagnosis of non-ST-elevation acute coronary syndrome (NSTE-ACS) who subsequently underwent coronary angiography were screened for eligibility. Patients who underwent coronary artery bypass grafting (*n* = 356), those who did not undergo percutaneous coronary intervention after coronary angiography (*n* = 219), and patients presenting with out-of-hospital cardiac arrest (*n* = 18) were excluded. Additional exclusion criteria were missing primary outcome data (*n* = 16), active infection (*n* = 25), active malignancy (*n* = 21), chronic inflammatory or autoimmune disease (*n* = 9), severe hepatic dysfunction (*n* = 8), hematologic disease affecting leukocyte subtypes (*n* = 11), and unavailable data required for calculation of the Naples or modified Naples scores (*n* = 46). After applying the predefined inclusion and exclusion criteria, a total of 921 patients with NSTE-ACS constituted the final study population.

The diagnosis of NSTE-ACS was established in accordance with the 2023 European Society of Cardiology (ESC) guidelines for the management of ACS [16]. A standard 12-lead ECG was obtained from all patients at admission. Baseline demographic characteristics, cardiovascular risk factors, comorbidities, angiographic and procedural findings, laboratory parameters, medication history, and in-hospital outcomes were retrospectively collected from institutional electronic medical records and national health registry systems. Venous blood samples were obtained from the antecubital vein at the time of admission before PCI. Routine biochemical parameters were analyzed using a fully automated chemistry analyzer, Beckman UniCel DXC 800 Synchron (Beckman Coulter Inc., Brea, CA, USA). High-sensitivity cardiac troponin I (hs-cTnI) levels were measured using a chemiluminescence immunoassay with the UniCel DXI 800 Synchron autoanalyzer (Beckman Coulter Inc., Brea, CA, USA). The estimated glomerular filtration rate (eGFR) was calculated using the Modification of Diet in Renal Disease formula [17]. Diabetes mellitus was defined as a documented previous diagnosis, current use of antidiabetic treatment, fasting plasma glucose ≥126 mg/dL, glycated hemoglobin ≥6.5%, 2-h plasma glucose ≥200 mg/dL during an oral glucose tolerance test, or random plasma glucose ≥200 mg/dL in the presence of symptoms. Hypertension, hyperlipidemia, heart failure, prior CAD/PCI history, and cerebrovascular events were defined according to documented medical history, previous diagnoses, or current use of relevant medications. Cerebrovascular events were defined as a clinically documented history of ischemic or hemorrhagic stroke supported by neurological evaluation and/or imaging findings. The study was conducted in accordance with the principles outlined in the Helsinki Declaration for biomedical research involving human subjects. The study protocol was approved by the local clinical research and ethics committee (No. 18-810, date: 23 October 2025). Given the retrospective nature of the study, the requirement for signed informed consent forms from individual participants was waived.

### 2.2. Naples and Modified Naples Score Calculation

The conventional Naples score was derived from four components: serum albumin, total cholesterol, NLR, and LMR. A point was allocated for each unfavourable value—serum albumin below 40 g/L (<4.0 g/dL), total cholesterol under 180 mg/dL, NLR of 2.96 or higher, and LMR below 4.44—while a value of 0 was given when the respective cutoff was not reached. Summing these four components yielded a conventional Naples score ranging from 0 to 4. The modified version of the score incorporated CRP as an additional parameter, adding one point for CRP levels above 2.56 mg/L and zero points for values at or below this threshold (Supplemantary Table S1) [15].

### 2.3. Coronary Angiography and Intervention

Coronary angiography was performed in all patients using a Siemens AXIOM Artis system (Siemens Healthcare, Erlangen, Germany), via radial or femoral access with 5- to 7-Fr diagnostic or guiding catheters. Antithrombotic management followed current guideline recommendations: acetylsalicylic acid was given as a 300 mg loading dose followed by 100 mg daily, combined with a P2Y_12_ inhibitor—either ticagrelor (180 mg loading dose, then 90 mg twice daily) or clopidogrel (600 mg loading dose, then 75 mg daily). Once the decision for PCI was made, unfractionated heparin was administered as an initial bolus of 70–100 IU/kg, with supplemental doses of 30–50 IU/kg given as needed for longer procedures.

The choice of vascular access, guiding catheter, stent type and size, use of pre-/post-dilatation balloons, thrombus aspiration, and glycoprotein IIb/IIIa inhibitor use was left to operator discretion. When administered, tirofiban was given as a 25 µg/kg bolus followed by a 12-h infusion at 0.15 µg/kg/min. During hospitalization, eligible patients were started on evidence-based secondary prevention therapy—including high-intensity statins, beta-blockers, and ACEis or ARBs—unless contraindicated. Two interventional cardiologists independently evaluated the coronary angiograms using a digital DICOM viewer (MedCom GmbH, Darmstadt, Germany). Coronary flow was graded according to the Thrombolysis in Myocardial Infarction (TIMI) flow classification. A final flow grade of 2 or lower in the absence of mechanical obstruction or significant residual stenosis was considered impaired coronary flow [18,19]. Thrombus burden was evaluated using the TIMI thrombus grading scale, ranging from grade 0, indicating no angiographic thrombus, to grade 5, indicating total vessel occlusion due to thrombus [20]. For each patient, the GRACE score was calculated using variables such as age, heart rate, systolic blood pressure, serum creatinine, Killip class, presence of cardiac arrest at admission, elevated cardiac biomarkers, and ST-segment deviation.

### 2.4. Statistical Analyses

Data and statistical analyses for this study were performed using the R Foundation for Statistical Computing (version 4.5.2, Vienna, Austria; URL: http://www.R-project.org). Categorical variables were expressed as numbers and percentages. Continuous variables were assessed for distributional characteristics using visual inspection and normality tests. Normally distributed variables were presented as mean ± standard deviation (SD), whereas non-normally distributed variables were presented as median with interquartile range. Comparisons between the low modified Naples score group (0–2) and the high modified Naples score group (3–5) were performed using the independent-samples *t*-test or Mann–Whitney U test for continuous variables, as appropriate. Categorical variables were compared using the chi-square test or Fisher’s exact test, where applicable.

Variables with a missing data rate of ≤3%, including N-terminal pro–B-type natriuretic peptide (NT-proBNP), GRACE score, and Killip score, were imputed using the MICE approach. Ten imputed datasets were generated using variable-specific imputation models, and pooled estimates were obtained according to Rubin’s rules. The primary endpoint of the study was in-hospital mortality. Univariable logistic regression analyses were performed to identify candidate predictors of in-hospital mortality. Multivariable logistic regression models were then constructed to evaluate the independent association of conventional Naples score and modified Naples score with in-hospital mortality. Because the conventional Naples score and modified Naples score share common components, these scores were entered into separate multivariable models to avoid multicollinearity and overadjustment. Odds ratio (OR), adjusted-OR, and 95% confidence interval (CI) were calculated for logistic regression analyses. Multicollinearity was assessed using the variance inflation factor (VIF). Model discrimination was evaluated using the C-index, corresponding to the area under the receiver operating characteristic (ROC) curve. Overall model performance was further assessed using the Brier score, likelihood-ratio chi-square statistic, Akaike Information Criterion (AIC), Bayesian Information Criterion (BIC), and McFadden R^2^. Nested model comparisons were performed using likelihood-ratio testing to assess the incremental prognostic contribution of GRACE score, conventional Naples score, and modified Naples score beyond the baseline clinical model. ROC curve analysis was performed to evaluate the discriminatory ability of conventional Naples score and modified Naples score for in-hospital mortality. Areas under the ROC curves (AUCs) were reported with 95% CI, and correlated AUCs were compared using the DeLong test [21]. Additional improvement in risk prediction was assessed using continuous net reclassification improvement, integrated discrimination improvement, and median improvement index [22]. Restricted cubic spline analysis was used to explore the dose–response relationship between conventional Naples score, modified Naples score, and in-hospital mortality. Finally, decision curve analysis (DCA) was performed to compare the net clinical benefit of the Naples and modified Naples scores in predicting in-hospital mortality, where a value of zero indicated no net benefit and values above zero reflected increasing benefit. All statistical tests were two-sided, with statistical significance set at an alpha level of 0.05.

## 3. Results

### 3.1. Baseline Characteristics, Procedural Data, and Outcomes

A total of 921 patients with NSTE-ACS were included in the final analysis. The mean age was 63.54 ± 11.51 years, and 573 patients (62.2%) were male. According to the predefined modified Naples score categories, 241 patients (26.2%) were classified into the low modified Naples score group (0–2), whereas 680 patients (73.8%) were classified into the high modified Naples score group (3–5).

**Figure 1:**
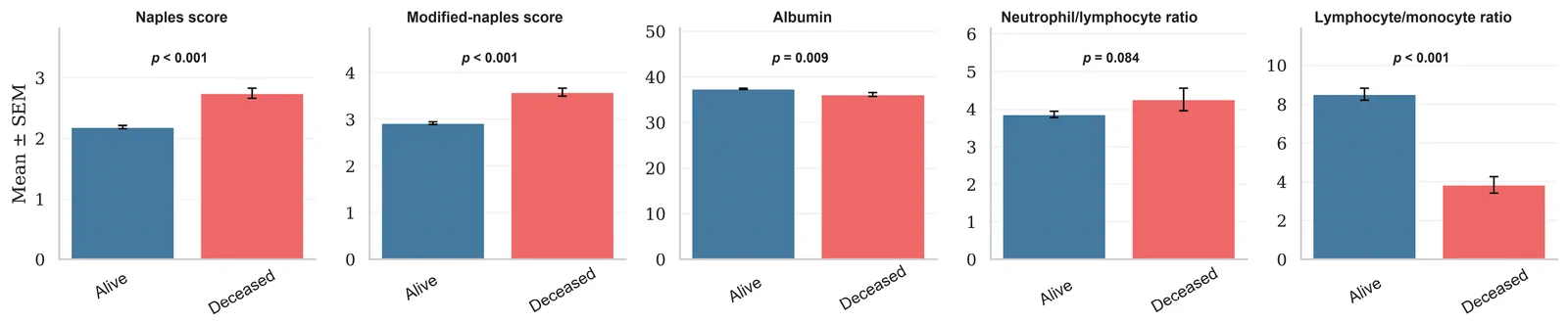
Score and inflammatory/nutritional markers according to in-hospital mortality.

Patients in the low modified Naples score group were slightly older (*p* = 0.024) and had higher GRACE score (*p* = 0.001), lower left ventricular ejection fraction (*p* = 0.022), and higher rates of diabetes mellitus (*p* = 0.031) and heart failure (*p* = 0.047). Several laboratory parameters also differed significantly between groups, including glucose, albumin, renal function parameters, lipid parameters, NT-proBNP, hs-cTnI, CRP, white blood cell count, platelets, NLR, and LMR (all *p* < 0.05). Regarding angiographic and procedural characteristics, TIMI thrombus burden differed significantly between groups (*p* = 0.022), whereas multi-vessel disease and other major procedural variables were comparable (Table 1). In-hospital mortality occurred in 79 patients (8.6%). Patients with in-hospital mortality had higher Naples score and modified Naples score values than survivors (2.75 ± 0.74 vs. 2.19 ± 0.73, *p* < 0.001; and 3.58 ± 0.76 vs. 2.91 ± 0.80, *p* < 0.001, respectively) (Figure 1).

### 3.2. Predictors of In-Hospital Mortality and Model Performance

In univariable logistic regression analysis, both Naples-based scores were significantly associated with in-hospital mortality. Each one-point increase in Naples score was associated with higher odds of in-hospital mortality (OR: 2.88; 95% CI: 2.06–4.02; *p* < 0.001). Similarly, each one-point increase in modified Naples score was associated with increased mortality risk (OR: 2.93; 95% CI: 2.14–4.01; *p* < 0.001). Among non-score variables, multi-vessel disease was strongly associated with in-hospital mortality (OR: 8.48; 95% CI: 5.16–13.92; *p* < 0.001). Higher GRACE score (OR: 1.02; 95% CI: 1.01–1.03; *p* < 0.001) and higher Killip score (OR: 2.07; 95% CI: 1.56–2.74; *p* < 0.001) were also significant univariable predictors. Lower left ventricular ejection fraction, lower eGFR, lower hemoglobin, lower lymphocyte count, and lower LMR were additionally associated with higher in-hospital mortality risk (Supplementary Table S2).

In multivariable logistic regression models, both modified Naples score and Naples score remained independently associated with in-hospital mortality after adjustment for baseline clinical variables and GRACE score. In the model including modified Naples score, modified Naples score was independently associated with in-hospital mortality (adjusted-OR: 3.85, 95% CI: 2.71–5.48; *p* < 0.001). In the corresponding model including conventional Naples score, Naples score was also independently associated with in-hospital mortality (adjusted-OR: 4.52, 95% CI: 3.05–6.70; *p* < 0.001). In these models, GRACE score and TIMI flow also remained significant predictors of in-hospital mortality (Table 2).

The baseline clinical model showed moderate overall performance. Adding the GRACE score improved model fit, and further inclusion of either Naples-based score provided additional improvement. The modified Naples score and conventional Naples score models showed similar performance across Brier score, likelihood-ratio chi-square, AIC, BIC, and McFadden R^2^ values, indicating comparable incremental prognostic contribution beyond the GRACE-adjusted clinical model (Table 3).

Discrimination and reclassification analyses demonstrated that the addition of Naples-based scores provided significant incremental prognostic information beyond the baseline clinical model. The model incorporating modified Naples score improved net reclassification by 74.1% (95% CI: 0.513–0.951; *p* < 0.001), integrated discrimination by 12.7% (95% CI: 0.090–0.165; *p* < 0.001), and median improvement by 8.1% (95% CI: 0.036–0.140; *p* = 0.001). Similarly, the model incorporating conventional Naples score improved net reclassification by 84.3% (95% CI: 0.641–1.046; *p* < 0.001), integrated discrimination by 12.5% (95% CI: 0.085–0.168; *p* < 0.001), and median improvement by 7.3% (95% CI: 0.058–0.136; *p* < 0.001). These findings indicate that both modified Naples score and conventional Naples score meaningfully improved risk stratification for in-hospital mortality, with overall comparable reclassification performance between the two scores (Table 4).

**Figure 2:**
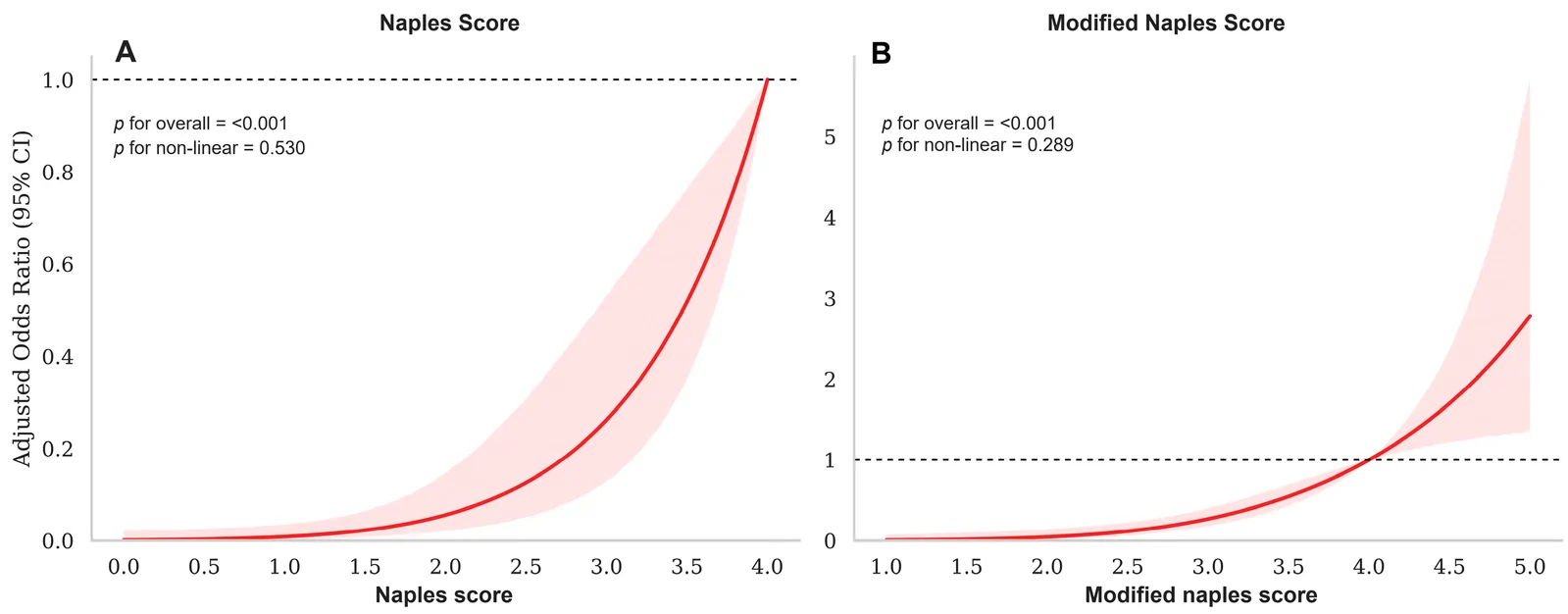
RCS analyses of the conventional (**A**) Naples score and (**B**) modified Naples score for in-hospital mortality.

Restricted cubic spline analyses showed significant overall associations between both Naples-based scores and in-hospital mortality. For Naples score, the overall association was significant (*p* for overall < 0.001), with no evidence of significant non-linearity (*p* for non-linearity = 0.530). Similar findings were observed for modified Naples score (*p* for overall < 0.001; *p* for non-linearity = 0.289), suggesting predominantly linear associations across the observed score range (Figure 2).

**Figure 3:**
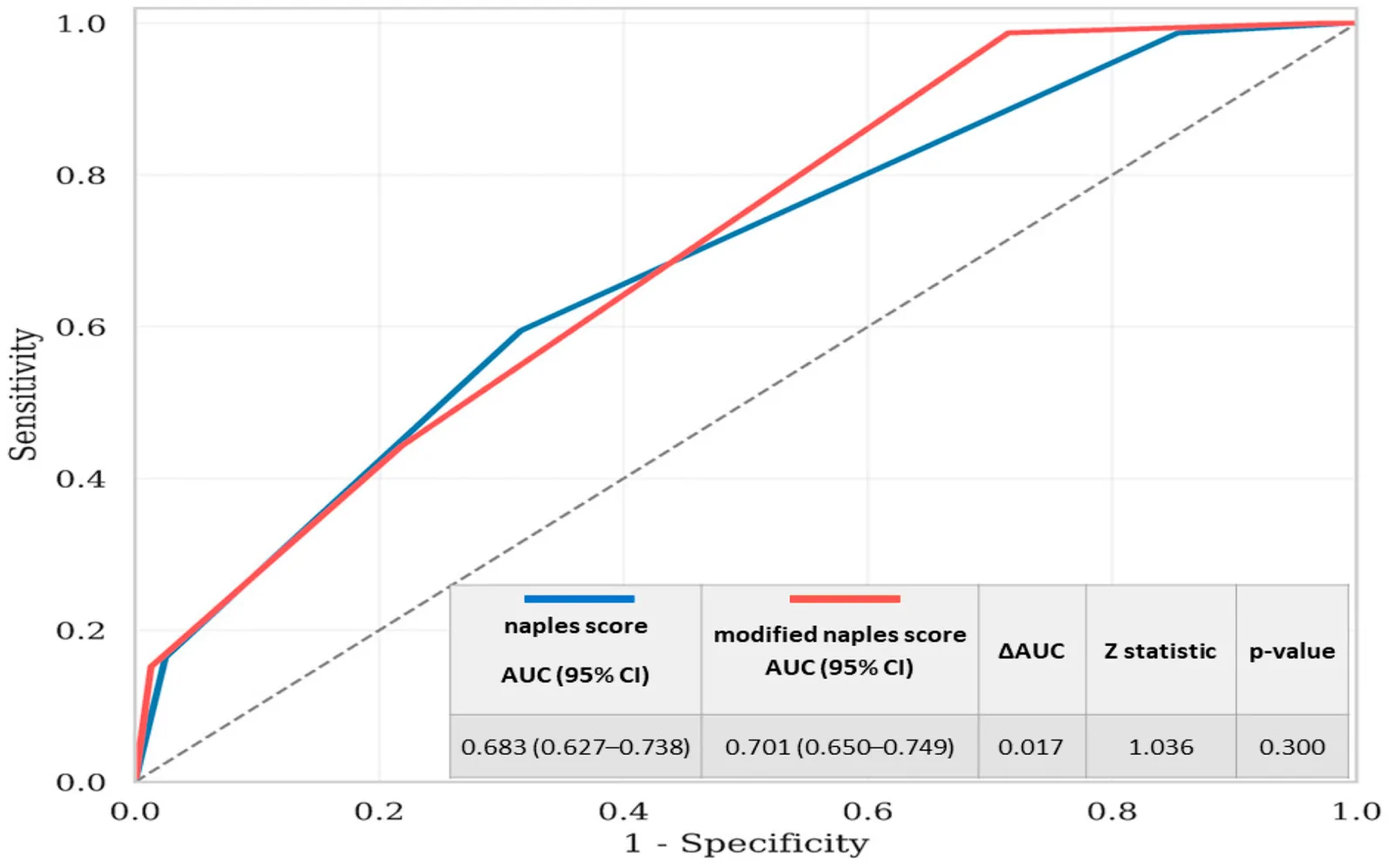
ROC analysis comparing Naples score and modified Naples score for in-hospital mortality. DeLong test was used for ∆AUC comparison.

**Figure 4:**
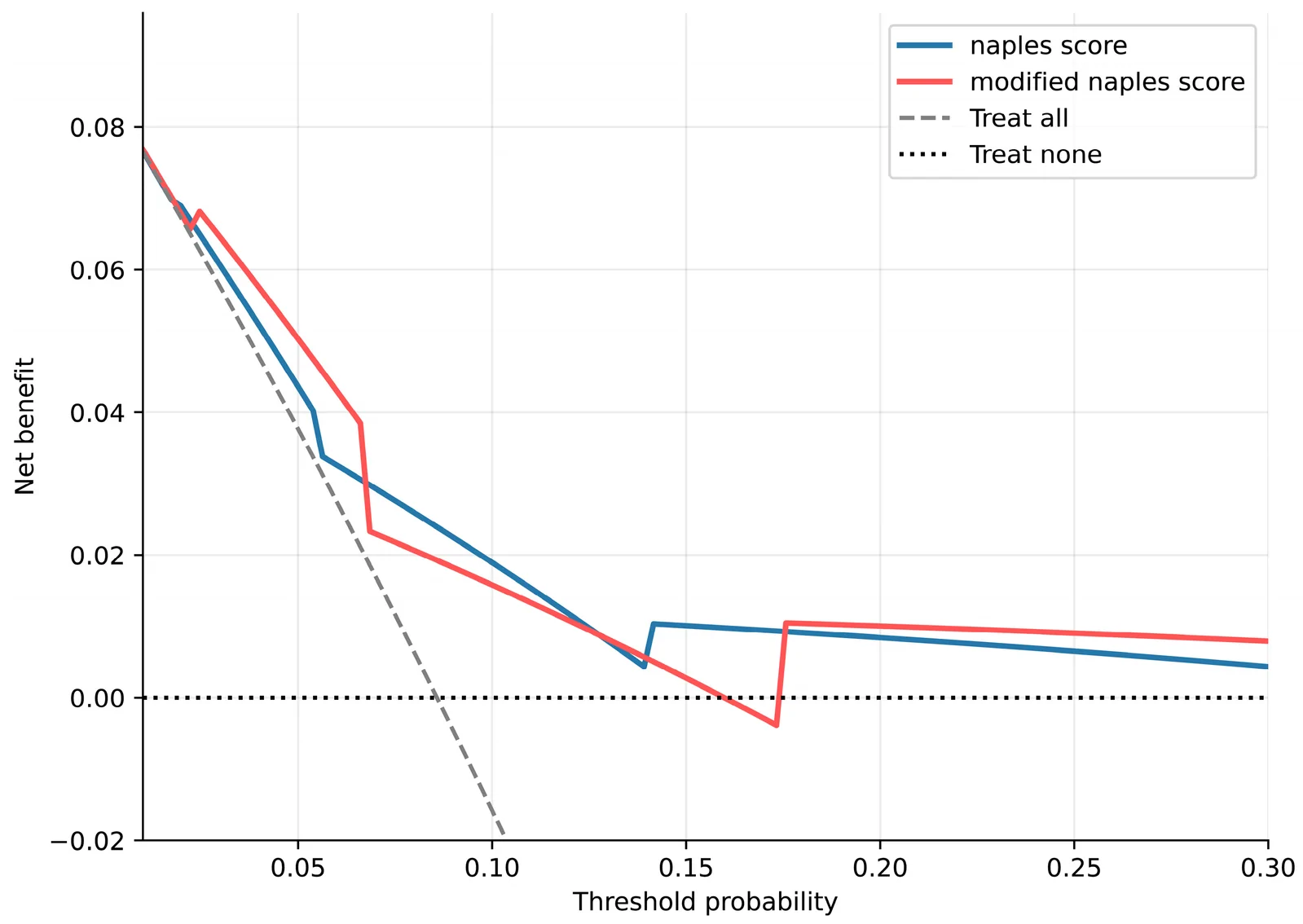
Decision curve analysis for in-hospital mortality.

In ROC analysis, the AUC for Naples score was 0.683 (95% CI: 0.627–0.738), whereas the AUC for modified Naples score was 0.701 (95% CI: 0.650–0.749). Although modified Naples score showed numerically higher discrimination, the difference between the two scores was not statistically significant on DeLong testing (ΔAUC = 0.017; Z = 1.036; *p* = 0.300) (Figure 3). Decision curve analysis showed that both the conventional Naples score and modified Naples score provided positive net clinical benefit across clinically relevant threshold probabilities compared with the treat-none strategy. However, neither score demonstrated a consistent superiority over the other in terms of net benefit (Figure 4).

## 4. Discussion

The main findings of this study can be summarized as follows: (i) Patients who died during hospitalization following PCI for NSTE-ACS had lower albumin, but higher CRP, Naples score, and modified Naples score values compared with those who were discharged with favourable outcomes. (ii) Both the Naples and modified Naples scores effectively predicted in-hospital all-cause mortality among NSTE-ACS patients undergoing PCI. (iii) The modified Naples score demonstrated discriminatory performance comparable to that of the conventional Naples score in identifying poor in-hospital prognosis. To our knowledge, this is among the first studies to directly compare the conventional Naples score and CRP-modified Naples score for in-hospital mortality risk stratification in patients with NSTE-ACS undergoing PCI.

The Naples score combines markers of systemic inflammation (NLR, LMR) with indicators of nutritional status (albumin, total cholesterol), both of which are increasingly recognized as important contributors to cardiovascular risk. Inflammatory cells and processes, including neutrophils and lymphocytes, contribute to atherosclerotic plaque instability and thrombus development by producing pro-oxidant and pro-inflammatory mediators, playing a significant role in the pathogenesis of ACS [23]. The chronic inflammatory state in the pathophysiology of CAD contributes to pathological plaque formation, linked to poor outcomes in ACS [24,25]. Additionally, unlike plasma protein biomarkers that require time for synthesis before showing a measurable rise, preformed blood cells can be released rapidly from the bone marrow into circulation. Stress-induced release of endogenous catecholamines and glucocorticoids triggers this rapid mobilization from bone marrow reserves shortly after the onset of chest pain. This rise in circulating cell counts may reflect underlying low-grade inflammation [26]. Population-based studies support this, linking higher monocyte counts to greater MACE risk [27]. This may relate to the dense monocyte/macrophage infiltration seen in vulnerable plaques with thin fibrous caps, suggesting that pre-existing monocytosis in AMI promotes heavier infiltration after plaque rupture and a more extensive thrombotic response [28]. Lymphocytes likewise contribute both to the acute inflammatory phase of AMI and to subsequent myocardial remodelling [6].

Laboratory-based hematologic tests provide quick and reliable results in recognizing on-going inflammation. Albumin, a major plasma protein synthesized by the liver and a negative acute-phase reactant, has been linked to low levels in advanced liver disease, malnutrition, hemodilution, and chronic systemic inflammation [29]. Notably, low albumin levels are common in ACS and CAD patients; in a large cohort, approximately 75% of patients had albumin levels <4.08 g/dL. Hypoalbuminemia is an independent predictor of new-onset heart failure and in-hospital mortality in patients with ACS [30]. In addition, serum albumin represents a key component of frailty indices, which have been closely linked to both short- and long-term mortality in cardiovascular patients undergoing percutaneous or transcatheter interventions [7,30,31,32].

Predictably, biomarkers derived from hematologic inflammatory indices have been highly effective and consistent in stratifying risk in ACS patients. For instance, elevated NLR was associated with an increased risk of CAD and greater severity [33], poor collateral development [34], cardiac dysfunction [35], and poor in-hospital and long-term outcomes after coronary intervention [2,36] and cardiac surgery [37]. Several studies have evaluated lymphocyte-based inflammatory indices for their prognostic value in patients with ACS [6,15,38]. Li et al. demonstrated that hematological inflammatory ratios such as NLR and MLR improve the predictive power of the GRACE score for long-term outcomes and risk classification in ACS patients undergoing PCI [38]. Similarly, in our study we showed that the Naples score and modified Naples score, which incorporate these parameters in addition to the GRACE score, provided better prognostic prediction.

In the present study, NLR, LMR, Naples score, and modified Naples score were all associated with in-hospital mortality risk. The in-hospital mortality rate in our cohort was 8.6%, which is broadly consistent with previous studies and registry data reporting in-hospital mortality rates of approximately 4.7% to 10.9% among patients with NSTE-ACS or ACS, particularly in higher-risk hospitalized populations [14,14,15,39]. However, biomarkers derived solely from bone marrow may not fully or consistently capture a patient’s inflammatory status [9]. A more accurate picture may instead emerge from combining these with plasma protein-based markers—particularly liver-synthesized, acute-phase-related markers such as albumin, CRP, or lipids. The modified Naples score, which integrates markers from both origins, may therefore better meet this need. Although several modified versions of the Naples score exist, the CRP-added form developed and validated by Genc et al. in 2576 STEMI patients showed better prediction of in-hospital mortality than the conventional Naples score [15]. In our study of NSTE-ACS patients, we found both the conventional Naples score and the CRP-modified Naples score to be similarly useful and comparable in performance. CRP is synthesized and released by hepatocytes in response to cytokines, particularly IL-6, with serum levels rising after ACS and peaking within 72 h after revascularization [40,41]. In addition, Matyas et al. showed that IL-6 and CRP were associated with worse ventricular remodelling and progression to heart failure after AMI [39]. In STEMI patients, elevated inflammatory biomarkers such as CRP have been linked to a higher risk of 90-day mortality, cardiogenic shock, and heart failure development [42]. Another study suggested that CRP may be considered a marker of the severity of the inflammatory response and may provide prognostic information regarding mortality risk [43]. This suggests that CRP may serve as a marker reflecting the severity of ischemia. Despite this evidence, CRP has not been incorporated into many of the inflammation-based indices discussed above; however, the modified Naples score addresses this limitation by including CRP as a component. Although we did not observe a significant difference between the conventional and modified Naples scores in our study, regression analysis revealed that CRP was closely associated with mortality and adverse outcomes, supporting its potential relevance in risk stratification for NSTE-ACS patients. Further studies with larger and more diverse patient populations are warranted to elucidate the comparative prognostic utility of these indices.

Our findings suggest that the Naples score reflects the combined prognostic impact of inflammatory and nutritional status in patients with NSTE-ACS. One apparently paradoxical finding was that patients in the lower modified Naples score group were older and had a higher burden of some comorbidities, but still had lower in-hospital mortality. This may be because the modified Naples score captures inflammatory and nutritional vulnerability rather than age or comorbidity burden alone. Patients with higher modified Naples score values had a more adverse biomarker profile, including higher CRP, lower albumin, unfavorable lipid parameters, and altered leukocyte-derived indices, which may reflect more pronounced systemic inflammation and impaired nutritional status during the acute phase of NSTE-ACS. Although the CRP-modified Naples score showed a numerically higher AUC than the conventional Naples score, this difference was not statistically significant, indicating comparable discriminative performance between the two scores. Therefore, the main clinical implication of our findings is that both Naples-based scores may provide additional prognostic information beyond the GRACE score and conventional clinical risk variables, rather than demonstrating clear superiority of the CRP-modified score. Unlike the TIMI and GRACE scores, which mainly focus on clinical and ischemic risk, Naples-based scores also incorporate inflammatory and nutritional parameters and may therefore offer a complementary perspective for early risk stratification. Given their simplicity and availability in routine practice, these scores may help clinicians identify patients who require closer monitoring or earlier intervention. Future prospective, multicenter studies are needed to validate these findings and to compare Naples-based scores with other established risk scores and inflammatory or nutritional indices in patients with NSTE-ACS.

Several limitations of our study should be acknowledged. First, its retrospective, single-center design carries an inherent risk of selection bias and limits our ability to draw causal conclusions. Second, our analysis was based solely on Naples score obtained at admission, and we did not assess how temporal changes in these biomarkers might relate to prognosis over time. Third, mid- and long-term follow-up outcomes were not available. Additionally, the retrospective nature of the study precluded separate assessment of individual MACE components. Finally, our findings are specific to the NSTE-ACS population studied and may not be generalizable to other patient groups, such as those with STEMI or chronic coronary syndrome. These limitations highlight the need for larger, prospectively designed studies incorporating more detailed longitudinal evaluation of clinical parameters to confirm and extend our findings.

## 5. Conclusions

In conclusion, both the conventional and modified Naples scores were significantly associated with in-hospital all-cause mortality in NSTE-ACS patients undergoing PCI. Although adding CRP did not markedly improve discriminatory performance over the conventional score, CRP itself remained strongly associated with mortality on regression analysis. By combining bone marrow-derived cellular indices with a hepatocyte-derived acute-phase marker, the modified Naples score may offer a more comprehensive reflection of patients’ inflammatory and nutritional status. These findings support the use of the Naples score, in both forms, as a simple and cost-effective tool for early risk stratification, though larger multicenter studies are needed to confirm its incremental prognostic value.

## Figures and Tables

**Table 1:** Baseline characteristics according to modified Naples score group.

Variable		Pooled	Low Modified Naples Score: 0–2 (*n* = 241)	High Modified Naples Score: 3–5 (*n* = 680)	*p*-Value *
Basic characteristics and admission parameters					
Age, years		63.5 ± 11.5	65.0 ± 11.7	63.0 ± 11.4	0.024
Gender, male, *n* (%)		573 (62.2)	148 (61.4)	425 (62.5)	0.764
Systolic blood pressure, mm Hg		132.4 ± 21.7	130.7 ± 21.6	133.0 ± 21.7	0.163
Diastolic blood pressure, mm Hg		78.3 ± 14.2	76.8 ± 13.7	78.9 ± 14.3	0.045
Heart rate, bpm		81.3 ± 15.7	83.9 ± 17.2	80.4 ± 15.1	0.005
GRACE score		120.7 ± 29.3	126.5 ± 29.9	118.8 ± 28.9	0.001
Left ventricular ejection fraction, %		49.7 ± 9.8	48.41 ± 10.08	50.12 ± 9.62	0.022
Length of stay, days		4.2 ± 1.9	4.3 ± 1.7	4.2 ± 1.9	0.461
Smoking status, *n* (%)					0.083
	Current	345 (37.5)	104 (43.2)	241 (35.4)	
	Former	242 (26.3)	54 (22.4)	188 (27.6)	
	No	334 (36.3)	83 (34.4)	251 (36.9)	
Diabetes mellitus, *n* (%)		438 (47.6)	129 (53.5)	309 (45.4)	0.031
Hypertension, *n* (%)		548 (59.5)	154 (63.9)	394 (57.9)	0.105
Cerebrovascular events, *n* (%)		65 (7.1)	17 (7.1)	48 (7.1)	0.998
Coronary artery disease, *n* (%)		388 (42.1)	111 (46.1)	277 (40.7)	0.150
COPD history, *n* (%)		60 (6.5)	19 (7.9)	41 (6.0)	0.316
Heart failure, *n* (%)		143 (15.5)	47 (19.5)	96 (14.1)	0.047
Hyperlipidemia, *n* (%)		337 (36.6)	94 (39.0)	243 (35.7)	0.365
Laboratory findings					
Glucose, mg/dL		169.4 ± 91.5	181.1 ± 91.4	165.3 ± 91.3	0.022
Albumin, g/L		37.3 ± 4.0	36.2 ± 4.0	37.7 ± 3.9	<0.001
Urea, mg/dL		40.9 ± 22.3	46.1 ± 27.3	39.0 ± 19.9	<0.001
Creatinine, mg/dL		0.85 [0.70–1.08]	0.90 [0.73–1.21]	0.85 [0.69–1.05]	0.015
eGFR, mL/min/1.73 m^2^		81.18 ± 25.85	75.91 ± 26.91	83.05 ± 25.22	<0.001
Sodium, mmol/L		137.7 ± 3.5	137.4 ± 3.6	137.7 ± 3.4	0.288
Potassium, mEq/L		4.34 ± 0.52	4.40 ± 0.52	4.32 ± 0.52	0.032
ALT, IU/L		27.1 ± 30.3	31.0 ± 39.3	25.7 ± 26.2	0.052
AST, IU/L		44.3 ± 53.3	52.3 ± 69.2	41.5 ± 46.1	0.026
Triglycerides, mg/dL		179.4 ± 137.1	170.6 ± 138.7	182.6 ± 136.5	0.249
HDL-C, mg/dL		42.6 ± 14.0	41.1 ± 10.4	43.1 ± 15.0	0.027
LDL-C, mg/dL		121.2 ± 35.2	114.7 ± 31.4	123.6 ± 36.2	<0.001
Total-C, mg/dL		185.5 ± 48.8	175.0 ± 45.1	189.3 ± 49.5	<0.001
NT-proBNP, pg/mL		957 [343–3450]	1515 [412–4559]	857 [318–3066]	<0.001
hs-cTnI, ng/L		1072 [231–3681]	1856 [273–5953]	874 [218–3242]	<0.001
CK-MB, ng/mL		24.69 ± 37.90	30.18 ± 45.04	22.74 ± 34.86	0.020
CRP, mg/L		7.6 [3.0–19.9]	18.3 [2.0–47.1]	7.1 [3.6–14.1]	0.010
WBC, ×10^3^/μL		10.21 ± 3.35	11.54 ± 3.94	9.74 ± 2.98	<0.001
Hemoglobin, g/dL		13.1 ± 2.0	12.9 ± 2.1	13.2 ± 2.0	0.020
Platelets, ×10^3^/μL		255.6 ± 78.2	273.8 ± 91.3	249.2 ± 71.9	<0.001
Neutrophil, ×10^3^/μL		7.18 ± 3.15	8.22 ± 3.86	6.81 ± 2.76	<0.001
Lymphocyte, ×10^3^/μL		2.33 ± 1.27	2.87 ± 1.48	2.15 ± 1.14	<0.001
Monocyte, ×10^3^/μL		0.54 ± 0.31	0.34 ± 0.24	0.60 ± 0.30	<0.001
Neutrophil/lymphocyte ratio		3.89 ± 2.65	3.59 ± 2.35	4.00 ± 2.75	0.026
Lymphocyte/monocyte ratio		3.6 [2.3–11.6]	10.3 [5.5–17.7]	2.9 [2.0–6.1]	<0.001
Naples score		2.23 ± 0.74	1.46 ± 0.55	2.51 ± 0.59	<0.001
Modified Naples score		2.97 ± 0.81	1.89 ± 0.32	3.36 ± 0.55	<0.001
Medications taken at the time of admission, *n* (%)					
Acetylsalicylic acid, *n* (%)		93 (10.1)	26 (10.8)	67 (9.9)	0.679
Clopidogrel, *n* (%)		12 (1.3)	6 (2.5)	6 (0.9)	0.091
ACEi or ARB, *n* (%)		79 (8.6)	21 (8.7)	58 (8.5)	0.930
Beta-blockers, *n* (%)		89 (9.7)	26 (10.8)	63 (9.3)	0.492
Statins, *n* (%)		137 (14.9)	41 (17.0)	96 (14.1)	0.278
Oral anticoagulants, *n* (%)		68 (7.4)	14 (5.8)	54 (7.9)	0.277
Angiographic and procedural features					
Culprit vessel, *n* (%)					
	LM	28 (3.0)	5 (2.1)	23 (3.4)	0.310
	LAD	391 (42.5)	107 (44.4)	284 (41.8)	0.477
	RCA	359 (39.0)	93 (38.6)	266 (39.1)	0.885
	LCx	231 (25.1)	54 (22.4)	177 (26.0)	0.265
Multi vessel disease, *n* (%)		121 (13.1)	24 (10.0)	97 (14.3)	0.089
Killip score, *n* (%)					0.433
	I	611 (66.3)	153 (63.5)	458 (67.4)	
	II	209 (22.7)	55 (22.8)	154 (22.6)	
	III	97 (10.5)	32 (13.3)	65 (9.6)	
	IV	4 (0.4)	1 (0.4)	3 (0.4)	
Balloon pre-dilatation, *n* (%)		781 (84.8)	204 (84.6)	577 (84.9)	0.939
Malignant arrhythmia, *n* (%)		14 (1.5)	4 (1.7)	10 (1.5)	0.767
Baseline TIMI flow, *n* (%)					0.565
	0	6 (0.7)	3 (1.2)	3 (0.4)	
	I	92 (10.0)	24 (10.0)	68 (10.0)	
	II	70 (7.6)	20 (8.3)	50 (7.4)	
	III	753 (81.8)	194 (80.5)	559 (82.2)	
Final TIMI flow, *n* (%)					0.780
	0-I	6 (0.7)	1 (0.4)	5 (0.7)	
	II	23 (2.5)	7 (2.9)	16 (2.4)	
	III	892 (96.9)	233 (96.7)	659 (96.9)	
No-reflow, *n* (%)		33 (3.6)	10 (4.1)	23 (3.4)	0.582
TIMI thrombus burden, *n* (%)					0.022
	0	67 (7.3)	18 (7.5)	49 (7.2)	
	I	97 (10.5)	30 (12.4)	67 (9.9)	
	II	294 (31.9)	94 (39.0)	200 (29.4)	
	III	282 (30.6)	60 (24.9)	222 (32.6)	
	IV	151 (16.4)	30 (12.4)	121 (17.8)	
	V	30 (3.3)	9 (3.7)	21 (3.1)	
GP IIb/IIIa inhibitor infusion, *n* (%)		33 (3.6)	8 (3.3)	25 (3.7)	0.798
Mechanical circulatory support, *n* (%)		18 (2.0)	5 (2.1)	13 (1.9)	0.793

Data are shown in *n* (%), median (interquartile range; 25th–75th percentiles), and mean ± SD. * A *p*-value of <0.05 was considered statistically significant. Abbreviations: ACEi, Angiotensin-converting-enzyme inhibitors; ALT, alanine transaminase; ARB, Angiotensin receptor blockers; AST, aspartate aminotransferase; CRP, C-reactive protein; eGFR, estimated glomerular filtration rate; HDL-C, high-density lipoprotein cholesterol; hs-cTnI, high-sensitivity cardiac troponin I; LDL-C, low-density lipo-protein cholesterol; NT-proBNP, N-terminal pro–B-type natriuretic peptide; Total-C, total cholesterol; WBC, white blood cell.

**Table 2:** Multivariable logistic regression models for in-hospital mortality.

Model	Variable	Adjusted-OR	95% CI	*p*-Value *
Model 1	Killip score	1.56	1.09–2.22	0.014
	NT-proBNP	1.00	1.00–1.00	0.121
	eGFR	1.00	0.99–1.01	0.838
	Left ventricular ejection fraction	0.99	0.97–1.02	0.648
	Hemoglobin	0.95	0.83–1.08	0.428
	Age	1.02	0.99–1.04	0.215
	TIMI flow	0.74	0.54–1.02	0.066
	Smoke	0.91	0.67–1.25	0.566
Model 2	Killip score	1.16	0.78–1.72	0.474
	NT-proBNP	1.00	1.00–1.00	0.230
	eGFR	1.00	0.99–1.01	0.920
	Left ventricular ejection fraction	1.00	0.97–1.03	0.985
	Hemoglobin	0.95	0.83–1.09	0.464
	Age	0.97	0.94–1.01	0.148
	TIMI flow	0.60	0.43–0.84	0.003
	Smoke	0.93	0.68–1.28	0.661
	GRACE score	1.03	1.01–1.04	<0.001
Model 3	Killip score	1.13	0.74–1.72	0.565
	NT-proBNP	1.00	1.00–1.00	0.047
	eGFR	1.00	0.99–1.01	0.720
	Left ventricular ejection fraction	1.00	0.97–1.03	0.805
	Hemoglobin	0.94	0.81–1.08	0.367
	Age	0.97	0.94–1.01	0.190
	TIMI flow	0.52	0.37–0.75	<0.001
	Smoke	0.84	0.60–1.18	0.327
	GRACE score	1.03	1.01–1.04	0.001
	Modified Naples score	3.85	2.71–5.48	<0.001
Model 4	Killip score	1.15	0.75–1.77	0.523
	NT-proBNP	1.00	1.00–1.00	0.027
	eGFR	1.00	0.99–1.01	0.827
	Left ventricular ejection fraction	0.99	0.96–1.02	0.534
	Hemoglobin	0.92	0.80–1.07	0.275
	Age	0.98	0.94–1.02	0.240
	TIMI flow	0.47	0.33–0.68	<0.001
	Smoke	0.85	0.61–1.20	0.359
	GRACE score	1.03	1.01–1.04	0.002
	Naples score	4.52	3.05–6.70	<0.001

* A *p*-value of <0.05 was considered statistically significant. Abbreviations: CI, confidence interval; OR, odds ratio.

**Table 3:** Model performance metrics.

Model	Brier Score	LR Chi-Square	df	LR *p*-Value *	AIC	BIC	McFadden R^2^
Model 1	0.075	39.07	8	<0.001	518.0	561.4	0.072
Model 2	0.072	50.13	9	<0.001	508.9	557.2	0.093
Model 3	0.064	117.63	10	<0.001	443.4	496.5	0.218
Model 4	0.064	118.72	10	<0.001	442.4	495.4	0.220

* A *p*-value of <0.05 was considered statistically significant.

**Table 4:** Discrimination and reclassification performance of the predictive models for in-hospital mortality.

	Goodness of Fit		NRI		IDI		Median Improvement	
	C-Index (95% CI)	*p*-Value *	Index (95% CI)	*p*-Value *	Index (95% CI)	*p*-Value *	Index (95% CI)	*p*-Value *
Model 1	0.707 (0.642–0.763)	<0.001	ref	-	ref	-	-	-
Model 2	0.706 (0.639–0.771)	<0.001	0.255 (0.020–0.500)	0.034	0.023 (0.007–0.041)	0.008	0.003 (−0.005–0.012)	0.531
Model 3	0.819 (0.767–0.865)	<0.001	0.741 (0.513–0.951)	<0.001	0.127 (0.090–0.165)	<0.001	0.081 (0.036–0.140)	0.001
Model 4	0.827 (0.779–0.872)	<0.001	0.843 (0.641–1.046)	<0.001	0.125 (0.085–0.168)	<0.001	0.073 (0.058–0.136)	<0.001

Model definitions: Model 1: baseline clinical model including Killip score, NT-proBNP, eGFR, left ventricular ejection fraction, hemoglobin, age, TIMI flow, and smoke. Model 2: Model 1 plus GRACE score. Model 3: Model 2 plus Modified Naples score. Model 4: Model 2 plus Naples score. * A *p*-value of <0.05 was considered statistically significant. Abbreviations: CI, confidence interval; IDI, integrated discrimination improvement; NRI, net reclassification index.

## Data Availability

Data are available on request due to privacy and ethical restrictions.

## Supplementary Materials

The following supporting information can be downloaded at: https://www.mdpi.com/article/10.3390/medicina62081573/s1. Table S1. Components and cut-off values of the conventional and modified Naples scores; Table S2. Univariable predictors of inhospital mortality.
