# Supplementary material for: Prognostic Value of Conventional and Modified Naples Scores for In-Hospital Mortality in Patients with Non-ST-Elevation Acute Coronary Syndrome
Source: Medicina (Kaunas). 2026 Aug 17;62(8):1573. doi: 10.3390/medicina62081573 (PMC13514995; doi:10.3390/medicina62081573)
Supplement: Supplementary file 1 [file medicina-62-01573-s001.zip › Supplemantary Table S1.pdf]

Supplementary Table S1. Components and cut-off values of the conventional and modified Naples scores

Component	Unfavorable cut-off value	Conventional Naples score	Modified Naples score	Point assignment
Serum albumin	<40 g/L (<4.0 g/dL)	Included	Included	1 point if below cut-off
Total cholesterol	<180 mg/dL	Included	Included	1 point if below cut-off
Neutrophil-to-lymphocyte ratio (NLR)	≥2.96	Included	Included	1 point if at or above cut-off
Lymphocyte-to-monocyte ratio (LMR)	<4.44	Included	Included	1 point if below cut-off
C-reactive protein (CRP)	>2.56 mg/L	Not included	Included	1 point if above cut-off
**Total score range**	—	**0–4 points**	**0–5 points**	**Higher scores indicate greater inflammatory/nutritional risk**

Abbreviations: CRP, C-reactive protein; LMR, lymphocyte-to-monocyte ratio; NLR, neutrophil-to-lymphocyte ratio.
Note: The modified Naples score uses the same four components as the conventional Naples score and additionally incorporates CRP. Each unfavorable component is assigned 1 point.
