# Supplementary material for: Prognostic Value of Conventional and Modified Naples Scores for In-Hospital Mortality in Patients with Non-ST-Elevation Acute Coronary Syndrome
Source: Medicina (Kaunas). 2026 Aug 17;62(8):1573. doi: 10.3390/medicina62081573 (PMC13514995; doi:10.3390/medicina62081573)
Supplement: Supplementary file 1 [file medicina-62-01573-s001.zip › Supplemantary Table S2.pdf]

Supplementary Table S2. Univariable predictors of inhospital mortality		
Variable	OR (95% CI)	P value
multiple vessel disease	8.48 (5.16–13.92)	<0.001
modified naples score	2.93 (2.14–4.01)	<0.001
naples score	2.88 (2.06–4.02)	<0.001
grace score	1.02 (1.01–1.03)	<0.001
killip score	2.07 (1.56–2.74)	<0.001
BNP	1.00 (1.00–1.00)	<0.001
Lymphocyte/monocyte ratio	0.88 (0.83–0.94)	<0.001
eGFR	0.98 (0.98–0.99)	<0.001
Left ventricular ejection fraction	0.96 (0.94–0.98)	<0.001
Hemoglobin	0.82 (0.74–0.92)	<0.001
age	1.04 (1.02–1.06)	<0.001
lymphocyte	0.65 (0.50–0.84)	<0.001
monocyte	3.01 (1.38–6.54)	0.005
Albumin	0.93 (0.87–0.98)	0.009
pre dilatatation	2.04 (1.19–3.51)	0.010
White blood cell	0.90 (0.83–0.97)	0.010
Urea	1.01 (1.00–1.02)	0.012
heart failure	1.98 (1.15–3.41)	0.013
taken oral anticoagulant	2.23 (1.12–4.45)	0.023
TIMI flow	0.72 (0.54–0.96)	0.028
smoke	0.75 (0.57–0.99)	0.040
